# Extensive vulvar and perianal langerhans cell histiocytosis with clinical improvement during low-dose thalidomide and topical therapy: a case report

**DOI:** 10.3389/fmed.2026.1969330

**Published:** 2026-10-09

**Authors:** Pan Zou, Xiao Mei Zhu

**Affiliations:** 1 Department of Dermatology, Union Hospital, Tongji Medical College, Huazhong University of Science and Technology, Wuhan, Hubei, China; 2 Department of Dermatology, Tongji Hospital, Tongji Medical College, Huazhong University of Science and Technology, Wuhan, Hubei, China

**Keywords:** BRAF V600E, case report, langerhans cell histiocytosis, perianal region, thalidomide, vulva

## Abstract

Langerhans cell histiocytosis (LCH) involving the female genital region is uncommon and can closely resemble chronic inflammatory, infectious, or neoplastic vulvar diseases. We report a case of a 35-year-old woman with a 6-month history of recurrent painful brownish-red plaques, nodules, erosions, and ulcerations that began in the perianal region and subsequently extended to the vulva. Topical fusidic acid only provided temporary symptomatic relief. Histopathological examination revealed epidermal erosion and crusting, acanthosis, elongated rete ridges, and a dense dermal and subcutaneous infiltrate containing numerous large histiocytes with pale eosinophilic cytoplasm and focal nuclear grooves. Immunohistochemical analysis revealed that the lesional cells exhibited positivity for S100, CD1a, and langerin and negative for HMB45, SOX10, ALK-1A4, and BRAF V600E. Broader molecular testing for MAPK-pathway alterations was not performed, and the lesion was therefore described as BRAF V600E-immunonegative rather than genetically BRAF/MAPK wild type. The pathological diagnosis was vulvar LCH, with clinically suspected involvement of the perianal region. Positron emission tomography/computed tomography (PET/CT) revealed an enlarged cervical lymph node, with no other abnormalities documented; its relationship to LCH could not be determined. Oral thalidomide (50 mg nightly) was subsequently administered while topical Pevisone was continued. Symptomatic improvement, particularly pain relief, was observed after 1 week, and further clinical improvement of the anogenital lesions was documented at 3 months. Because both therapies were used concomitantly, the independent contribution of thalidomide could not be determined. This case underscores the diagnostic challenges associated with anogenital LCH and documents short-term clinical improvement during a low-dose thalidomide-containing regimen without establishing thalidomide monotherapy efficacy. It also highlights the necessity for biopsy, comprehensive systemic evaluation, and ongoing follow-up.

## Introduction

Female genital tract involvement is an uncommon manifestation of Langerhans cell histiocytosis (LCH), and the published evidence consists mainly of case reports, small case series, and retrospective studies (1, 2). Reported presentations have been categorized into four groups: disease confined to the female genital tract; genital disease that precedes subsequent systemic involvement; genital involvement accompanied by cutaneous or oral lesions; and genital involvement as a component of established multisystem LCH (1).

Because vulvar and perianal lesions can resemble inflammatory, infectious, or neoplastic disease, diagnosis may be delayed (1, 2). This study presents a case of extensive vulvar and perianal LCH characterized as BRAF V600E-immunonegative on immunohistochemistry, without broader MAPK-pathway molecular testing. The patient exhibited short-term improvement following a regimen containing low-dose thalidomide. The findings underscore the importance of diagnostic biopsy, comprehensive systemic assessment, and tailored treatment approaches.

## Case presentation

A 35-year-old female patient presented with recurrent, painful lesions in the perianal and vulvar regions persisting for six months. The initial eruption manifested as a fingernail-sized erythematous patch in the perianal area, without any apparent precipitating factor. Over time, surface erosion and ulceration developed, accompanied by pain. The patient applied topical fusidic acid and Pevisone ointment, which provided temporary symptomatic relief; however, the lesion recurred, progressively enlarged, and extended from the perianal to the vulvar region. As the condition progressed, the eruption became more extensive, characterized by brown-red patches and plaques, nodular thickening, moist erosions, crusting, and ulceration. The associated pain intensified, significantly interfering with sitting and other daily activities.

Her medical history includes a diagnosis of thyroid carcinoma, which was treated with a thyroidectomy in 2014, followed by regular administration of levothyroxine. She reported no notable psychosocial concerns and had no relevant family history or known genetic disorders. A dermatological examination identified extensive brown-red lesions affecting the vulva, perineal skin, and perianal region. The vulvar lesions were characterized by irregular patches and infiltrated plaques, with multiple areas exhibiting nodular thickening, surface erosion, crust formation, and ulceration. Similar erythematous and eroded plaques were noted around the anus. The presence of plaques, nodules, erosions, crusts, and ulcers was documented in pretreatment clinical photographs, as depicted in Figures 1A,B.

**Figure 1:**
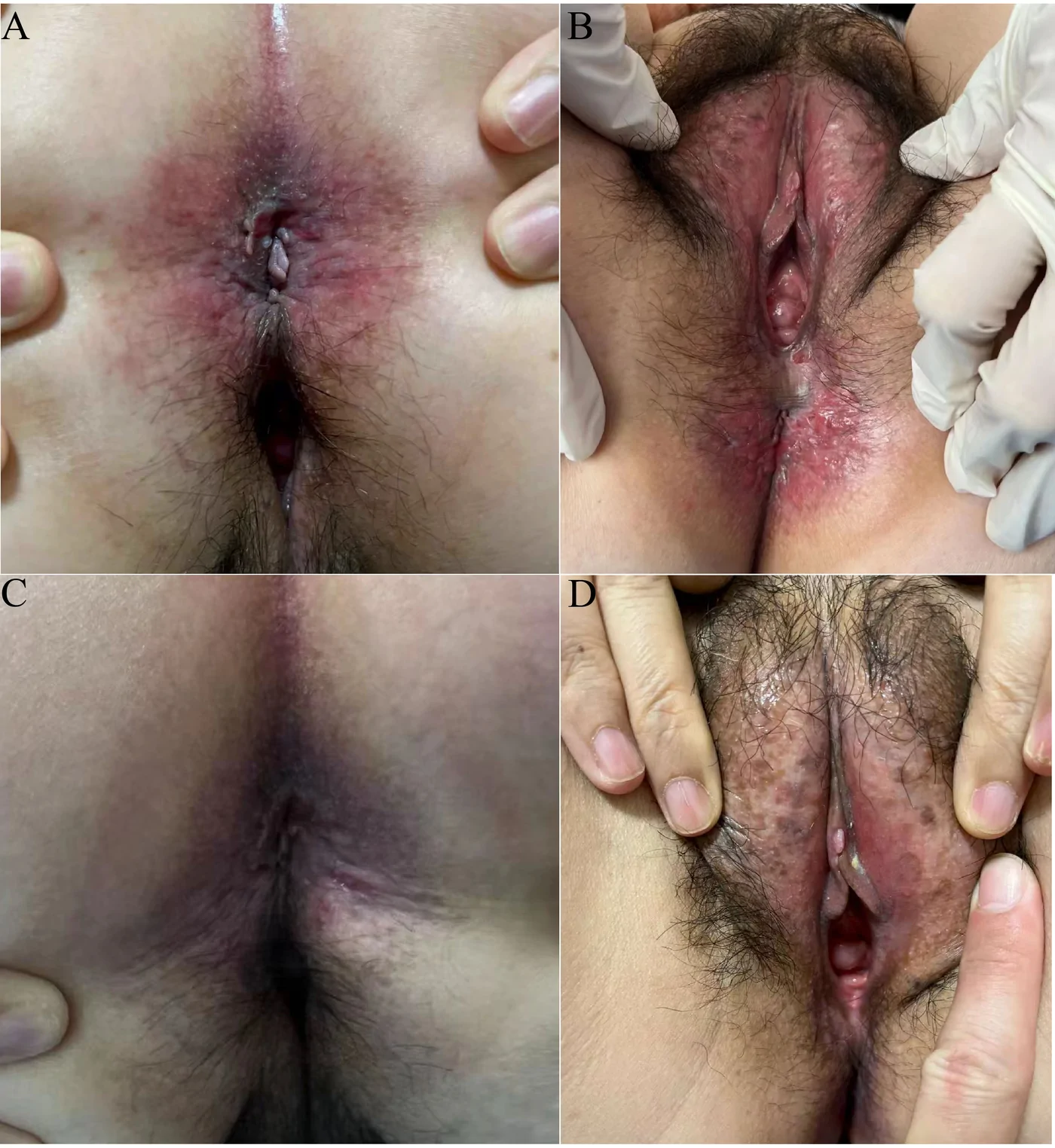
Clinical appearance before and after treatment. **(A)** Pretreatment perianal lesions showing brown-red plaques with erosions and ulceration. **(B)** Pretreatment vulvar and perineal lesions showing extensive brown-red infiltrated plaques, erosions, crusting, and ulceration. **(C)** Perianal lesions at 3 months showing similar clinical improvement with residual erythematous-brown plaques. **(D)** Vulvar and perineal lesions at 3 months after initiation of oral thalidomide (50 mg nightly) with continued topical Pevisone, showing marked reduction in erythema, erosion, ulceration, and infiltration, with residual brown-red erythema and plaques.

A biopsy was conducted on an active lesion. Hematoxylin and eosin staining indicated surface erosion accompanied by crust formation, epidermal acanthosis, and elongation of the rete ridges. A dense inflammatory infiltrate, primarily composed of histiocytes mixed with lymphocytes, extended from the dermis into the subcutaneous tissue. Many histiocytes appeared large and round, with weakly eosinophilic cytoplasm and enlarged nuclei, and some cells displayed focal longitudinal nuclear grooves. Representative low- and high-power findings are presented in Figure 2.

**Figure 2:**
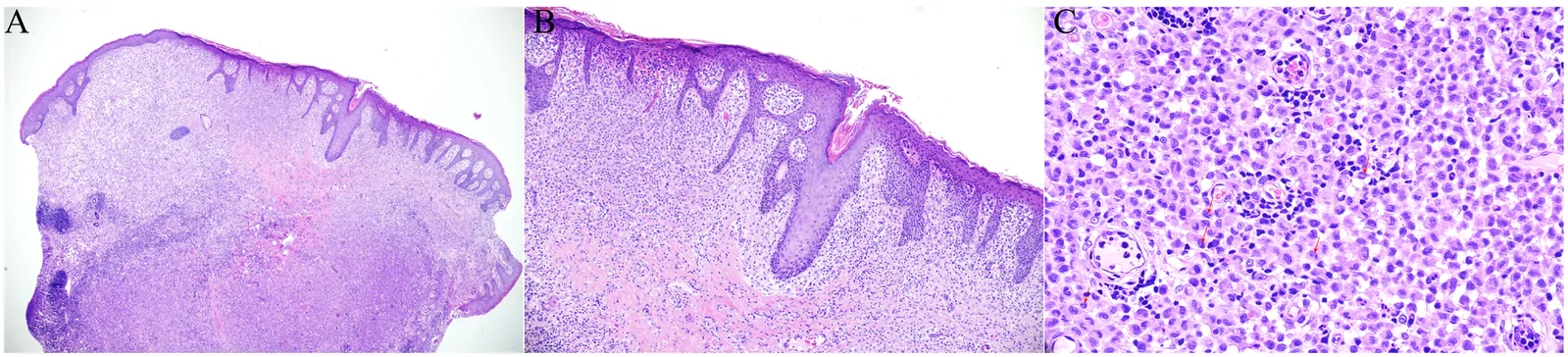
Histopathological findings of the vulvar lesion. **(A)** Surface erosion and crusting with epidermal acanthosis and elongation of the rete ridges (H&E,  × 40). **(B)** Dense dermal infiltrate composed predominantly of histiocytes admixed with lymphocytes (H&E,  × 100). **(C)** High-power view showing representative Langerhans cells with pale eosinophilic cytoplasm and characteristic longitudinal nuclear grooves (arrows) (H&E,  × 400).

Immunohistochemical analysis demonstrated that the lesional cells were positive for S100, CD1a, and langerin and negative for HMB45, SOX10, and ALK-1A4. BRAF V600E immunohistochemistry was negative, with appropriate staining of the positive control (Figure 3). Based on the histological and immunophenotypic findings, the pathological diagnosis was vulvar LCH. The perianal lesions were clinically considered part of the same disease process because they preceded and subsequently extended to the vulvar region and exhibited similar clinical morphology; however, no separate biopsy of the perianal lesions was performed, and their involvement was therefore not histologically confirmed.

**Figure 3:**
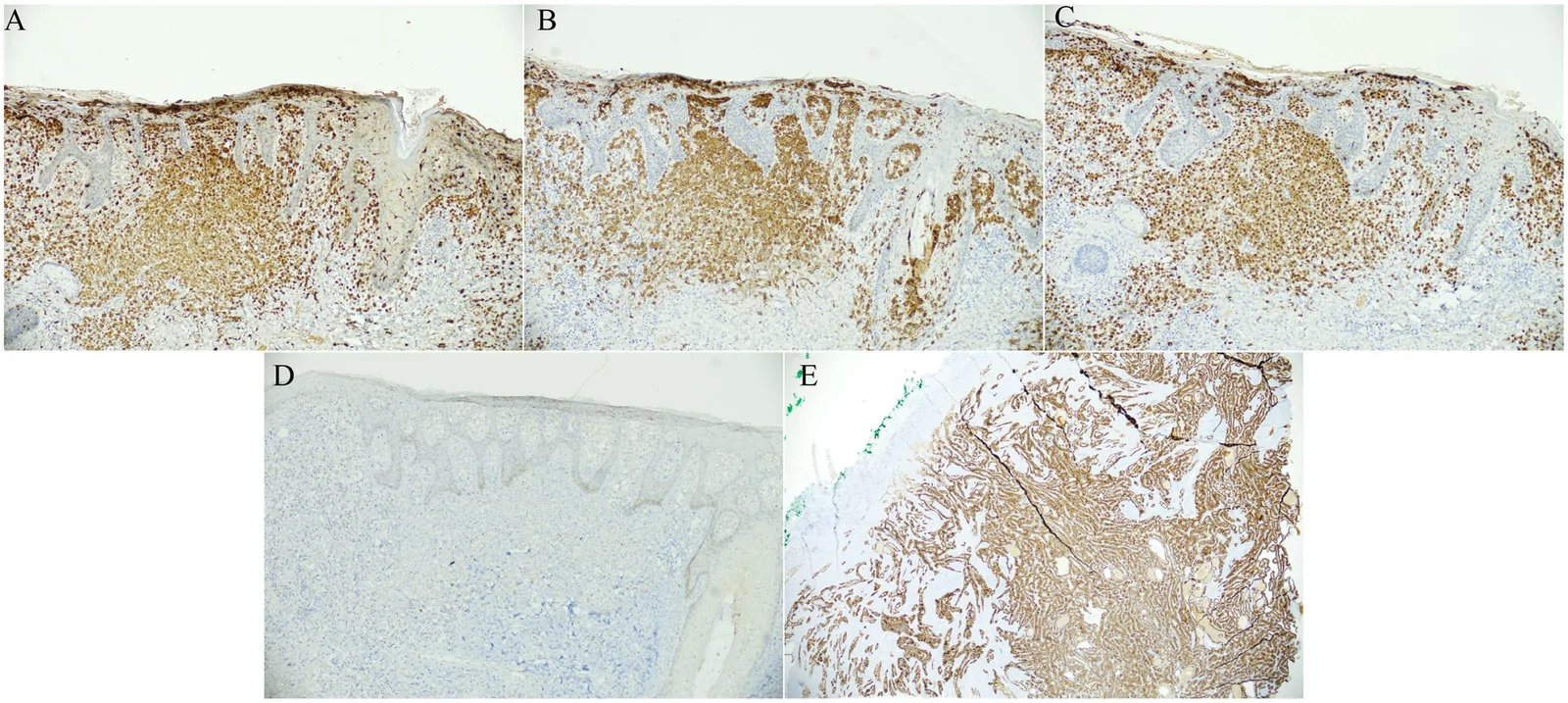
Immunohistochemical findings. The lesional cells showed positive staining for **(A)** S100, **(B)** CD1a, and **(C)** langerin, whereas **(D)** BRAF V600E staining was negative. **(E)** Positive control for BRAF V600E immunohistochemistry. Original magnification: **(A–E)** × 100.

Following the diagnosis, PET/CT was performed for systemic staging and revealed an enlarged cervical lymph node, with no other abnormalities documented. No further evaluation of this lymph node was performed at the initial assessment. During subsequent telephone follow-up, the patient reported that a cervical lymph node ultrasound performed at her local hospital showed no obvious abnormality. No FNAC or biopsy was subsequently performed. Therefore, although no persistent abnormality was reported on follow-up ultrasonography, histopathologic involvement of the cervical lymph node was not assessed. PET/CT was the principal documented systemic staging examination; detailed results of additional investigations, including complete blood count, liver and renal function tests, dedicated bone assessment, pulmonary assessment, and other organ-specific evaluations, were not available in the records for this retrospective report. Before thalidomide initiation, the patient had used topical Pevisone, a combination of triamcinolone acetonide and econazole nitrate, once to twice daily, with only limited improvement. Oral thalidomide was subsequently initiated at 50 mg nightly while topical Pevisone was continued. After 1 week, the patient reported marked relief of pain, representing early symptomatic improvement. At the 3-month follow-up, clinical examination and photographs showed reduction in erythema, erosion, ulceration, and infiltrative lesions, although residual brown-red erythema and plaques remained. Thalidomide was continued at 50 mg nightly without dose modification or discontinuation. No treatment-related adverse events were reported during the available follow-up. (Figures 1C,D). The patient reported that the pain, which had previously interfered with sitting and daily activities, was markedly relieved after treatment, and she perceived clear improvement in the anogenital lesions. The key events in the patient's clinical course are summarized in Figure 4.

**Figure 4:**
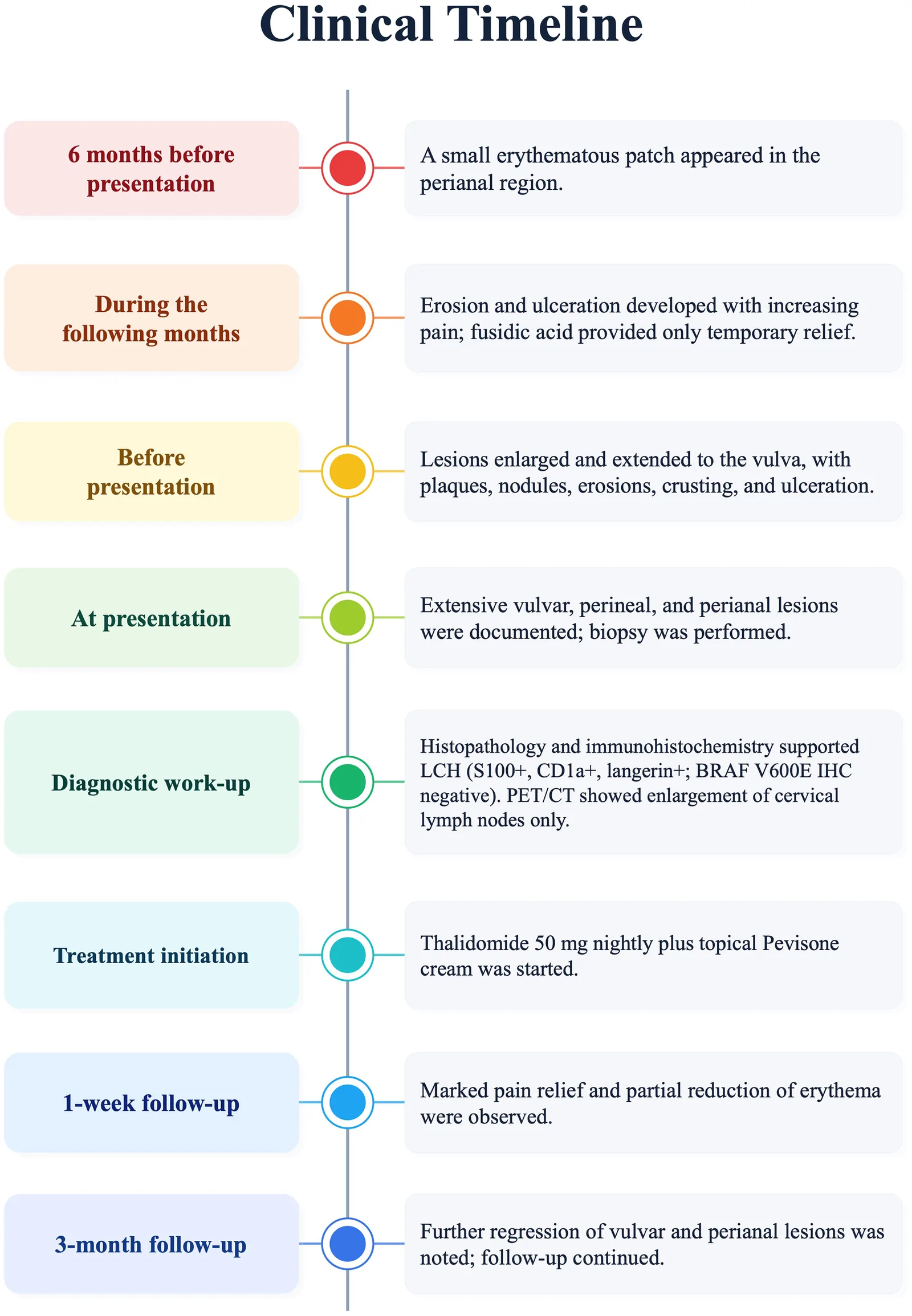
Relative clinical timeline.

## Discussion

### Diagnostic challenge of anogenital LCH

Anogenital LCH is diagnostically challenging because its clinical appearance overlaps with inflammatory, infectious, and neoplastic disorders. It may present with papules, plaques, nodules, erosions, crusting, ulceration, or variable pigmentation, with symptoms including pain, pruritus, dyspareunia, dysuria, and discomfort during sitting or walking (1–3). In this patient, progression from a small perianal erythematous patch to extensive painful vulvar and perineal plaques, nodules, erosions, and ulcers illustrates the nonspecific presentation and the importance of timely biopsy when lesions progress or respond incompletely to empirical therapy.

The clinical differential diagnosis of chronic erosive or ulcerative anogenital lesions is broad. Inflammatory disorders such as eczematous dermatitis, lichen sclerosus, erosive lichen planus, and pemphigus vegetans may present with erythema, crusting, erosions, fissures, or vegetating plaques. Infectious conditions, including genital herpes, syphilis, genital tuberculosis, and superficial bacterial or candidal infections, may show overlapping clinical features. Neoplastic disorders such as extramammary Paget disease, vulvar squamous cell carcinoma, and melanoma should also be considered in persistent or progressive lesions (4–10). Progressive infiltration, nodular thickening, persistent ulceration, and an incomplete response to empirical therapy constitute practical indications for conducting a timely biopsy instead of resorting to repeated topical treatments.

### Histopathologic confirmation and molecular interpretation

The diagnosis of LCH necessitates the presence of compatible morphology and a characteristic immunophenotype. Lesional cells typically exhibit pale or weakly eosinophilic cytoplasm, along with irregular, grooved, folded, or reniform nuclei. However, ulceration and the associated inflammatory infiltrate may obscure these diagnostic features. The principal defining markers are CD1a and langerin/CD207, while S100 is sensitive but less specific. The electron microscopic demonstration of Birbeck granules is no longer required when morphology and immunohistochemistry provide a definitive diagnosis (11, 12). In this case, large histiocytes with focal nuclear grooves and coexpression of S100, CD1a, and langerin established the diagnosis. Negative HMB45 and SOX10 staining argued against melanoma, while the CD1a/langerin-positive phenotype supported LCH over other histiocytic or dendritic-cell mimickers.

BRAF V600E immunohistochemistry was negative. However, this finding does not exclude LCH or establish a genetically wild-type MAPK pathway, because BRAF V600E is present in only a subset of cases and alternative alterations may involve MAP2K1, non-V600E BRAF variants, BRAF deletions or indels, and other MAPK-pathway genes (13, 14). As broader sequencing was not available, the lesion was more accurately described as BRAF V600E-immunonegative rather than MAPK-wild-type.

Systemic evaluation is required after diagnosis because genital LCH may be isolated, coexists with lesions elsewhere, or precedes multisystem disease (15). In this patient, PET/CT revealed an enlarged cervical lymph node. Its size and detailed radiologic characteristics were unavailable, and no initial ultrasonography, FNAC, or biopsy was performed; although subsequent patient-reported ultrasonography showed no obvious abnormality, the relationship of the node to LCH remains uncertain. In addition, detailed results of other systemic investigations were unavailable. Accordingly, this case cannot be definitively classified as disease confined to the anogenital region.

### Treatment of vulvar and female genital tract LCH

No standardized treatment algorithm exists for vulvar or female genital tract LCH, and published evidence is largely case based. Management ranges from observation or local therapy for limited lesions to systemic therapy for extensive, symptomatic, recurrent, function-limiting, or extracutaneous disease (1–3, 12). Therefore, treatment should be individualized according to lesion extent, symptoms, functional impairment, recurrence, systemic staging, and morbidity of the proposed intervention.

Observation or conservative local treatment may be reasonable for small, limited, cutaneous, or mucosal lesions. Local excision can be both diagnostic and therapeutic for a discrete lesion; however, extensive surgery is poorly suited to diffuse vulvar and perianal plaques because of the risks of pain, scarring, anatomic distortion, sexual dysfunction, and wound-healing difficulty. Therefore, historical reports of partial or radical vulvectomy should not be interpreted as support for aggressive surgery as routine first-line therapy (1, 2, 12). Radiotherapy has been used in selected cases, although local toxicity is an important concern for this sensitive anatomical site.

Systemic therapy is generally considered for extensive, symptomatic, recurrent, multifocal, function-limiting, or extracutaneous diseases. Current adult LCH recommendations include cytarabine or cladribine among systemic options, selected according to disease distribution, previous therapy, and patient factors (12). BRAF or MEK inhibition is relevant when an actionable MAPK alteration is identified, particularly in refractory or multisystem diseases; however, most targeted therapy data are derived from systemic LCH or mixed histiocytic disorder cohorts rather than isolated genital diseases (16, 17).

### Low-dose thalidomide in the present case

Thalidomide is one of the most frequently reported systemic treatments for predominantly cutaneous or genital LCH, although evidence remains limited to case reports and retrospective reviews. Its potential activity may be related to its immunomodulatory and anti-inflammatory effects, including cereblon-dependent protein regulation and modulation of cytokine pathways (18, 19). A literature review proposed thalidomide as a possible first-line or maintenance treatment for primary vulvar LCH, and a later case described resolution with 100 mg daily; however, recurrent disease has been reported, and durable control cannot be assumed (2, 3, 15).

In our patient, we prescribed thalidomide 50 mg nightly, a lower dose than the 100 mg daily regimen reported in some adult vulvar cases, together with topical Pevisone cream. We observed marked pain relief within 1 week and further visible regression over the following 3 months. Because Pevisone was continued throughout this period, its contribution cannot be excluded and the observed improvement cannot be attributed to thalidomide alone. The response is therefore best interpreted as short-term clinical improvement associated with a thalidomide-containing regimen rather than evidence of thalidomide monotherapy efficacy.

Thalidomide may be attractive when mucocutaneous lesions are too extensive for excision but do not otherwise justify intensive chemotherapy. Its convenience must be balanced against peripheral neuropathy, sedation, constipation, thromboembolic risk, and teratogenicity, particularly in women of reproductive potential (18, 19). Therefore, the lowest effective dose, periodic neurological and symptom assessments, and continued reassessment of disease extent are appropriate when treatment is prolonged.

### Strengths and limitations

The principal value of this case is the combination of a difficult anogenital presentation and an early response to a comparatively low-dose thalidomide-containing regimen. The simultaneous presence of plaques, nodules, erosions, crusting, and ulcers illustrates why clinical inspection alone may not distinguish LCH from other common inflammatory, infectious, or malignant conditions. The important limitations of this study include incomplete systemic staging, lack of histologic confirmation of the perianal lesions, absence of broader MAPK genotyping, concomitant use of topical Pevisone, limited thalidomide-specific safety monitoring, and follow-up limited to 3 months.

## Conclusion

Vulvar and perianal LCH should be considered in cases of chronic anogenital plaques, nodules, erosions, or ulcers that recur, progress, or respond incompletely to routine treatment. Timely biopsy and recognition of the characteristic S100-positive, CD1a-positive, and langerin-positive histiocytic infiltrate are central to diagnosis. Systemic assessment is required because genital disease may be isolated or accompanied by LCH at other sites. In this patient, oral thalidomide 50 mg nightly combined with continued topical Pevisone was associated with rapid symptomatic relief and visible clinical improvement over 3 months; because both treatments were used concomitantly, the independent effect of thalidomide cannot be determined. Continued follow-up is necessary to assess durability, recurrence, and treatment tolerance.

## Data Availability

The original contributions presented in the study are included in the article/Supplementary Material, further inquiries can be directed to the corresponding author.
